# Foreign

**Published:** 1841-09-11

**Authors:** 


### FOREIGN.

*Cases of Hepatic Abscess, in which exploration and puncture were adopted; with Clinical remarks*, by Dr. MURRAY, Inspector of Hospitals, Calcutta.

1*st.*—*Case of abscess of the liver, punctured.* By Dr. MORTIMER, Surgeon of the General Hospital.—The subject of the case was a seaman, aged 39, who had been about two years in India. He was admitted with the usual symptoms of acute hepatitis of some days' standing, which had, by account, succeeded an attack of bowel complaint.

The more urgent symptoms, after a time, appeared to yield in a great measure to the usual treatment; but the mercury used, although it affected his mouth, did not produce ptyalism, nor did he convalesce as he would have done had the disease been overcome.

The constitutional symptoms generally considered as indicative of abscess, although observable, were rather obscurely marked; and notwithstanding a good deal of general fulness, extending from one hypochondrium to the other, there was no particular spot in which local examination afforded unequivocal evidence of the existence of matter.

By the new exploring needle, however, having been introduced at two inches in a right line from the junction of the cartilages of the 7th and 8th ribs, a few drops of purulent matter were discharged through the tube of the instrument at its handle. The external opening was then enlarged, and a common trocar introduced through the canula of which about eight ounces of very thick healthy pus were evacuated.

The patient expressed himself considerably relieved by the operation, but this was the only benefit derived from it; and the case terminated fatally two days afterwards.

*Post-mortem appearances.*—On opening the abdomen, the liver was found stretching entirely across, and occupying both hypochondria: the stomach being altogether concealed and pushed backwards by the left lobe. There was no adhesion of the liver anteriorly to the peritoneal lining of the abdomen, excepting for about a quarter of an inch around the puncture, where there appeared a recent deposit of lymph.

It was found that the purulent matter discharged through the opening made before death had been contained in an abscess situated nearly in the middle of the right lobe. The size of the cavity appeared to have contracted considerably, and was separated by a septum of parenchymatous substance of about half an inch in thickness, from another and much larger abscess situated superiorly, and occupying the greater part of the upper and convex portion of the right lobe, which had pushed the diaphragm considerably upwards. There was another circumscribed abscess, larger than either of the other two, situated below and partly behind that first noticed, occupying the whole of the inferior part of the lobe. The contents of the last appeared to be retained within their cavity at the lower boundary merely by peritoneal covering of the viscus, somewhat thickened, and adhering strongly to the contiguous viscera and upper part of the right kidney, which were all so united together that they could not be separated. There was also an abscess in the middle of the left lobe of about the same size as the one first mentioned.

All the abscesses contained thick healthy-looking purulent matter.

2*d.*—*Three cases of hepatic abscess, in which the exploratory needle was used.* By Dr. MOUAT, 13th Light Dragoons.—Sergeant Damon Derrick, 4th (King's own) regiment; in India two years. Was attacked with acute hepatitis in the early part of last August, (1839,) for which he was bled three times, had leeches to his side twice, three blisters, calomel, purgatives, &c. with but little relief. The pain continued, though in a mitigated degree, and the side swelled, accompanied with colliquative stools and sweats, and every sign of hepatic abscess, when it was determined to puncture the liver.

On the 11th of August the exploratory needle was introduced posteriorly at the angle, and between the 7th and 8th ribs, to the depth of two inches and a half, but without detecting pus. However, we found no inconvenience ensue from the puncture, and the second day after the operation the abscess apparently burst into the intestines, and he gradually recovered from that period. He never experienced any bad symptom from the introduction of the exploratory needle; he thinks, on the contrary, that it rather did him good.

Other two men of the same regiment, private Jessey Austen and William Leg, were operated on about the same time in a similar manner, and experienced no bad consequences from the introduction of the needle. Leg was twice punctured: the first operation failed to reach the cyst; but the second puncture did, when a trocar was afterwares introduced, and a large abscess evacuated. He was better for some days, but then became worse, with an increase of hectic symptoms, and he died. On dissection it was found he had other abscesses:—the first puncture had healed, and the second, leading to the cyst of the abscess, had united it to the pleura. So that, as far as the operation was concerned, it was safe, and unattended with a single untoward consequence.

The same also may be said of Austen. His liver was punctured in two places without finding matter, yet he died with abscess in both lobes; when it was found that the punctures from the exploratory needle had healed, without producing any bad effect.

I was consulted in all these three cases, and I am fully satisfied as to the safety of puncturing the substance of the liver by the exploratory needle, which, I believe, I was the first to use and bring into notice at Bangalore.*

3*d.*—*Summary of the case of private Andrew Killagher, His Majesty's 39th Regiment.* By Assist.-Surgeon M'Grigor.—This man, æt. 33, sixteen years in the service, of turbulent character and irregular habits, was admitted into the hospital at Bellary, in the beginning of July 1839, with a *relapse* of acute hepatitis, and put under active antiphlogistic treatment, without its affecting the arrest of the disease. At the end of a fortnight he still complained of pain and weight in the epigastrium and right side; and, along with this, he had severe constitutional fever, which had reduced him so much that he lay supine, and seemed scarcely able to speak. Pulse 104; bowels irregular.

On the 22d of July I called Dr. Davidson, the superintending surgeon, into consultation, when it was agreed to puncture the liver, to which the patient willingly consented, from the prospect of its affording him relief.

I entered the trocar near the margin of the ribs, and close also to the ensiform cartilage; and as I continued to feel resistance, I pushed it in to its whole length; but on withdrawing the stilette blood only came out.

Much to our satisfaction, however, the patient expressed himself greatly relieved by the operation, and he continued from that time to improve.

I left the canula in the wound till the following night, with a view to prevent the escape of blood into the cavity of the peritoneum, and excite adhesion between this membrane and the liver; when, there being much pain around the puncture, and a feeling of stiffness of the abdomen, it was removed.

The wounded vessels in the liver had ceased to bleed; and there was no further hæmorrhage. The abdomen was fomented, a dose of morphia given, and the patient afterwards passed a very fine night.

Next morning we found him in raptures at the relief he had obtained. He had no pain nor uneasiness; but he had got a slight diarrhœa. Pulse 90.

By the 28th his improvement was so great that all were astonished. He said himself he was sure he would have died but for the operation; and certainly at the time I introduced the trocar I thought him in a very bad way.

Common simple treatment, chiefly alterative, aperient and dietetic, only was employed afterwards, and he gradually improved, but did not get quite well, his liver remained enlarged and heavy, his feet became slightly œdematous, and he was transferred to the Depôt, at Poonamalle, as an invalid, from whence he was sent to England for a change of climate.

4*th.*—*Case of Hepatic Abscess cured by early puncture.*—By Dr. Everhard, Asst.-Surg. H. M. 54th Regiment.—Private James M'Eldoon, H. M. 54th Regt. æt. 38; twelve years in India; generally healthy, but lately of intemperate habits, was admitted into the hospital 10th November, 1839, complaining of severe pain in the right hypochondrium, increased by inspiration or pressure, which he says came on three days ago, and has gradually increased since. Has severe headache; pulse full; skin hot; bowels open; urine high coloured.

Ft. V. S. ad effectum; R. Calomel gr. v.; Pulv. Jalap ʒiss.; spoon diet.

[Various remedies, chiefly mercury, leeches, and purging employed.]

20th.—Swelling more prominent, with feeling of fluctuation. There is evidently no adhesion between the liver and abdominal parietes. He is emaciated, and has sense of weight and oppression in the epigastrium.

Contin. Cataplasm.

* Though the operation of exploring deep seated abscesses had been previously practised in England, to Dr. Mouat is due the credit of introducing and recommending the practice in this presidency, and we believe in India.

22d.—The Deputy Inspector (Dr. Murray) visited the hospital this morning, and after examination of this man, thrust a trocar into his liver through the epigastrium, without waiting to make any preparatory operation to induce adhesion between the parts. About a pint and a half of thin brown matter came out on withdrawing the stilette, and the canula was left in the wound. The man looks pale, and has become weak. Pulse soft; skin cool; bowels freely open.

The canula was tied in by a bandage round the body, a bit of lint put into the orifice, and a large warm poultice over all. To have some light fish for dinner, and half a pint of beer; with tea and toast.

*Vespere.*—Has got great relief from the operation. Matter oozes out from the abscess by the canula.

R. Acet. Morphiæ. gr. ss. h.s.s.

23d.—Had a good night—doing well.

*Subsequent History.*—For a few days after the operation, about two ounces of unhealthy greenish bilious matter came away, after which the discharge became gradually more healthy (occasionally bilious) and less; but did not entirely cease until the 19th February following. As the liver continued enlarged and hard after the evacuation of the abscess, hydriodate of potash was given with advantage.

At present (10th March) his appetite and general health are good; he sleeps well, and he has recovered his strength. He has no uneasiness in the side, though the liver is attached to the abdominal parietes, where it is punctured, and feels somewhat enlarged. There was never any effusion of matter into the cavity of the peritonæum, as adhesion soon took place after the puncture. The operation is considered to have saved this patient's life.

5*th.*—*Abstract of a case of Hepatic Abscess, explored and punctured.* By Dr. Maclean.—Private John Gorman, H. M. 55th Regiment, aged 33, was admitted into the General Hospital under Dr. Mortimer on the 8th of February, 1840, with fever, cough, difficulty of breathing, mucous expectoration, and daily rigors at noon. He had been ill for four days previously to admission, and ascribed the cause of his illness to cold caught while on guard at night.

He did not complain particulary of uneasiness in the hepatic region or epigastrium till the 21st of February, when a general fulness in the latter was observable. On the 22d he felt very weak. On the 25th the fulness of the epigastrium and oppression of breathing were increased, and his countenance became anxious; pulse 84. On the 27th it is reported that he had again chills for several nights, followed by free perspiration; pulse 100. On the 29th he was transferred to the 54th Hospital under Dr. Everhard. On the 2d of March his liver was explored and punctured by Dr. Murray, who made the following memorandum at the time on the case.

Being informed by Dr. Everhard that he had got a patient in a dangerous state transferred to his hospital, suffering from pectoral symptoms with obscure manifestations of suppuration in the liver, I went to consult with him on the case.

I found the liver extending nearly three inches into the epigastrium towards the umbilicus, tender to touch, but not so much as to preclude examination. The right rectus muscle was more tense than the left, or rather it became so on attempting to examine the tumour, as if to screen it from pressure, which Mr. Twining gives as a characteristic symptom of central abscess of the liver. The patient had many shivering fits about three weeks ago, and now has profuse cold perspirations at night (*hectic fever,*) with a considerable puriform deposit in the urine; but there is no fluctuation perceptible in the enlarged viscus. Decubitus on the back or left side is extremely oppressive. He has a frequent tickling cough and great difficulty of breathing, with sense of weight in the hepatic region; his legs and body are œdematous; he has no appetite; great thirst; pulse 120 and intermittent; prostration of strength great.

Finding him suffering so much, and evidently in a very dangerous way, and considering the general as well as local symptoms decidedly indicative of existing suppuration, I pushed a trocar into the liver where it protruded into the epigastrium; but only a little blood flowed on withdrawing the stilette. Not satisfied with this exploration, I pushed the new exploratory instrument into the liver behind the middle of the side, between the eighth and ninth ribs, when, to our satisfaction, pus flowed; not, however, through the tube of the instrument, but by the side of it, apparently from my havig gone beyond the abscess.

I then withdrew the explorer, and introduced a large sized flat trocar, by which eight or nine ounces of thick curdy pus were evacuated. When the evacuation was nearly completed, a gurgling of air took place through the canula, apparently from the action of the diaphragm, and a cork was then fitted to the canula, (which was retained *in situ*) with directions to take it out at midday and in the evening, to allow accumulated matter to escape. A bit of sticking-plaster was applied over the orifice of the first puncture in the epigastrium.

After this, the patient's breathing, and his decubitus on the back, were somewhat relieved; and he said he felt altogether better. He complained of thirst, for which he was ordered small quantities of lemonade, and the following mixture.—

℞ Liq. Ammon. Acet. ℥v.; Spt. Æther Nitr. ʒij.; Syrup Simpl. ℥ij; Aquæ ℥xxx. (M.—℥iij. 3tiâ q. q. hora.)

Although his bowels were free, he was ordered a purging enema in the evening; and a grain of Acet. Morph. at bed-time. A large poultice to be applied over the hepatic region and side. Spoon diet; and congee.

3d March, *Mane.*—Says he did not sleep, and that he has not slept for many nights, but that he feels better this morning. The canula was withdrawn last night, and a tent of lint introduced instead. A little thick matter is discharged at each dressing. The urine is now clear. Pulse 100; skin warm and moist; bowels open; thirst less.

Contin. mistura. To have an egg and one pint of beer, with spoon diet.

*Vespere.*—Slept a good deal during the day; breathing easier; has no uneasiness in the site of the puncture in epigastrium; pulse 100; skin less clammy.

5th.—Did not rest so well. No discharge from the side this morning. The urine again deposits a thick yellow sediment; great depression and anxiety; pulse 120, and rather full; skin clammy. Dr. Murray made another exploratory puncture into the liver on the right side of the epigastrium, without finding pus; but a quantity of serous fluid was evacuated from the cavity of the abdomen, on withdrawing the canula out of the liver.

The side to be well fomented, and afterwards poulticed.

℞ Enema; R. Tinct. Opii; Tinct. Hyosciami, aa. M. L.; Aq. Puræ j. h. s. s.

He gradually sank, and died on the 13th.

*Dissection, seven hours after death.*—Previously to opening the body, the exploratory instrument was introduced into the liver, near the end of the eleventh (floating) rib, when thin yellow pus issued freely through its canal, shewing it had entered an abscess.

Œdematous swelling of the hands, feet, and ancles, with emaciation of the arms, legs, and thighs, abdominal enlargement, distension of the right hypochondrium, bulging of the ribs of the right side, and an ulcerative sloughy state of the wound, constituted the external appearances.

On dividing and turning back the abdominal parietes, it was found that firm adhesion, of recent formation, had taken place between it and the liver, where the two punctures were made in the epigastric region; and attentive examination could not detect any mark of inflammation in the substance of the gland around the points punctured; the cicatrization of it was perfect. The right lobe of the liver extended (was apparently pushed down) to within an inch of the crest of the illeum and umbilicus; and the left lobe nearly reached to the spleen.

It was found that the exploratory instrument which before dissection was pushed into the liver, had entered a large distinct abscess situated in the right side of the concave surface of the gland, which had very narrowly escaped being penetrated in the explorattion made on the 5th instant. Its area was considerably larger than a man's fist, and it contained upwards of a pint and a half of thick yellow greenish pus.

Immediately above this abscess was the empty contracted sac of the one opened and evacuated on the 2d of March; the inner surface of it had a dark gangrenous appearance, which extended throughout the course of the wound.

At the centre of the upper convex part of the liver was a third distinct abscess, *the largest of all*, containing nearly three pints of matter, which seemed not only the chief cause of the projection of the organ beyond the ribs, by its pushing it downwards, but also of the projection of the diaphragm into the right cavity of the chest: it pushed the diaphragm as high up as the fourth rib. The upper part of the walls of this abscess adhered extensively to the diaphragm; as did those of the lowest abscess to the cellular substance and other parts above the right kidney.

There was no adhesion between the liver and the colon or stomach.

The anterior part of the right lobe (where the two punctures in the epigastrium were made) and the left lobe were somewhat enlarged, but their substance did not appear otherwise unhealthy.

The gall bladder was contracted, and of a pale colour.

The right cavity of the thorax was full of darkish serum,—it contained at least five pints; and the lung of that side was collapsed, compressed into a surprisingly small size, non-crepitant, and perfectly unserviceable.

The left lung, heart, and large blood-vessels, presented no change from health.

From the state of the liver and contents of the left cavity of the thorax, there was no doubt as to the cause of death. The event was probably neither accelerated nor retarded by the punctures made in the former: it was judged that the patient was past recovery by any human means at the time they were made.

*Clinical Remarks by Dr. Murray.*—We have derived much information of an interesting and instructive kind from this unfortunate but important case [of Gorman.]

Three days subsequently to making the punctures on the 2d instant, the purulent discharge ceased, when great increase of dyspnœa, anxiety, and restlessness, supervened: and the liver was felt projecting still farther beyond the ribs all across the side.

The hectic fever, weight in the hepatic region, and sense of suffocation on decubitus on the back and left side, continued; the latter symptom was much more prominent than I ever witnessed in any case of simple hepatic disease; and was owing, as appeared on dissection, to the hydrothorax. The case was altogether distressing and alarming, and the prognosis most unfavourable.

In the hope of being able to reach another abscess, or to re-open the one I had before penetrated in a more depending position, and thereby afford relief to the patient, after making a preparatory puncture with a lancet through the integuments into the enlarged liver, about an inch and a half more to the right and lower than the first puncture (*i. e.* a little below the cartilaginous junction of the ninth to the eighth rib,) I inserted a trocar in the direction of the centre of the diaphragm, but without coming to any matter.

The operation did not appear to give much pain, (*the preparatory puncture with the lancet rendering the entrance of the trocar easy*,) and it was not followed by any bad effects; but we did not deem it advisable to institute any further exploration at this time.

Very little blood came from the liver, but on withdrawing the canula, a quantity of serous (*ascitic*) fluid escaped through it from the cavity of the peritonæum, indicating that no adhesion then existed between the liver and abdominal parietes at that part.

The details of the case, and post-mortem examination, show, first, that as no bad consequences resulted from making the punctures, danger need not be apprehended from pushing a trocar into the parenchymatous substance of an enlarged liver; secondly, that the punctures were the means of causing adhesion between the liver and abdominal parietes; thirdly, that the man's life, if it was not eased and prolonged, at any rate was not shortened by the operations; and, *lastly*, that even had I succeeded on the 2d instant in reaching all the abscesses to evacuate them, so great was the *quantum* of disease in the liver and right lung, that there would have been no chance of the patient's recovery.

It has been customary for medical officers to mark with a sort of *exultance* the unexpected discovery of an abscess in the liver after death, as if thereby showing that death must have been the inevitable result of any treatment in such cases; but, hereafter, I shall rather be inclined, *in general*, to consider such a discovery as a reflection upon their discrimination and practice.

There is at present too great reluctance on the part of most practitioners to explore enlarged livers, even when there are strong characteristic symptoms of existing abscess, from apprehension of danger in the operation. A *deterring* story is told here of a patient once dying from hæmorrhage in consequence of a trocar having been pushed into his liver; but I can call to mind seventeen cases within the last few years, wherein I performed this operation without any bad consequences; by which six of the patients were recovered, and are alive at this day I believe.

I consider, that, *with a good anatomical and pathological knowledge of the region in our mind's eye*, to enable us to avoid the large hepatic vessels, the gall-bladder,* the colon, and the stomach; there is abundance of evidence to authorize us, nay that it is our bounden duty, to explore the liver, without hesitation or delay, in most cases where pathognomonic symptoms of abscess in it exist, and the disease is interfering seriously and prejudiciously with the functions of the organ, and with the general health of the patient.

By early accurate diagnosis, and active constitutional and local (*preventive*) treatment, a favourable termination may very often be happily brought about in hepatic diseases without the necessity of operative procedure; but when abscess has once formed, we know how little advantage is to be expected from persisting in the use of mercury or any other medicine: therefore let the question be fairly put—Does not the trocar, with a well regulated diet, hold out a better prospect of success?

The case under consideration was at first under the care of my friend Dr. Mortimer, who I must mention was about to explore the liver when the man was transferred to the 54th hospital, where the Doctor continued to take a warm interest in him to the last, and kindly conducted the *Autopsia.*

After having seen this dissection, I would hereafter explore to a greater extent any analagous case; and I am, moreover, of opinion, that all our punctures should be made from the abdominal cavity—entering the trocar or explorer under the edge of the cartilages of the seventh, eighth, or ninth ribs, as circumstences may indicate. We may often indeed get nearer to the abscess through one of the intercostal spaces; and I think primary exploration may sometimes be advantageously made in this situation by a very minute flat canular instrument; but, from not having seen any patient recover where the matter was evacuated in this direction (through the diaphragm, from finding that the action of the fibres of the diaphragm impedes the free discharge of the matter, somewhat like a valve; from observing that air sometimes enters the wound when made here; and from considering that the opening is not so dependent through the walls of the thorax as when made through the abdominal parietes; I beg to recommend the latter mode in all cases; and I must also say that I would prefer a long flat trocar to any other instrument, as the stilette can be withdrawn occasionally during the operation to ascertain if any abscess has been penetrated; and the canula can be left in *situ* afterwards, if thought desirable.

When the abscess is central, or situated in

* A tumid gall-bladder projecting at the epigastrium, may be distinguished from an abscess of the liver, by a peculiar induration continuously surrounding the latter which is not present in the former, it being a soft, elastic circumscribed tumor, the result of over distension from fluid in a natural cavity.

the upper (convex) part of the liver, it will require much confidence and boldness in the practitioner to operate effectually; but what arduous operation does not? The example of perseverance set us by Surgeon Wilkins, of the 41st regiment, (mentioned at page 480, Vol. I. of this journal,) where he introduced the long trocar for puncturing the bladder in order to reach the abscess, by which means he cured his patient, should not be lost sight of.

It seems to me only necessary to draw the attention of medical officers to the general fatality of hepatic abscess, and to excite reflection thereon, to carry conviction to their minds of the advantage of the practice I have been endeavoring to establish. I am by no means anxious, however, that my views should be hastily adopted, as nothing is more injurious than the reception of any proposition without scrupulous examination:—if it be false, this perpetuates error; if true, it abridges its utility by leaving the principle insufficiently investigated and explained, and thus abridging its application.*

---

*On the Treatment of Uterine Hæmorrhage; with a recommendation for the use of a Tourniquet in such cases.* By William Pretty, M. R. C. S.—In casual conversation with the late Mr. Walford, teacher of midwifery in Aldersgate Street School, upon the treatment of uterine hæmorrhage after delivery, he suggested to me the application of pressure by means of the tourniquet as certain of success; and such was his confidence in this means, that he emphatically declared that no woman ought to die of uterine hæmorrhage; and that any practitioner of midwifery losing a woman from this cause, ought to suffer the punishment due to the crime of manslaughter.

From having had some experience in these frightful cases, and more especially from its occurrence twice in my own domestic circle, each time producing all but fatal syncope, notwithstanding every care was taken to guard against such a lamentable result, by the attendance of a kind and talented physician-accoucheur; and knowing the great difficulty of successfully treating and managing such cases, they have been a subject particularly interesting to me. It is in cases of flooding after delivery that I have found the use of the tourniquet so highly satisfactory, and that I strongly recommend its employment to all accoucheurs. I know not how far the members of the profession generally are acquainted with the use of this apparatus for such a purpose, but finding those with whom I have conversed ignorant of its great value as a means of saving life, I have thought that my humble testimony to its merits might not be without its utility.

To witness the death of a woman from uterine hæmorrhage after delivery is an appalling sight, and a sad calamity, which it becomes every medical man to do all in his power to avert. I think also that we ought to aim at something more than the preservation of life; I mean, if possible, to prevent those many and great evils which generally follow perilous cases of flooding—such as protracted debility, incapability to nurse the offspring, and its consequent suffering from the want of natural nourishment; causing wet nurses to be sought for, who cannot always be found or easily paid for, and are often very troublesome besides: there is here also a large amount of domestic anxiety and trouble only fully known to those who have experienced it. I believe that the tourniquet will not only arrest the violent and large discharges of blood from the uterus, but will likewise prevent that slow draining away of it, which, without producing syncope, is oftentimes the cause of great exhaustion and a long convalescence. Its use will also diminish the amount of after pains, as exemplified in a case to be related, of a woman confined of her seventh child. It will likewise relieve the practitioner of much bodily exertion, and materially abridge the period of watchfulness; for feeling assured that his patient is safe, all painful anxiety is removed from his mind.

Many are the means employed to prevent or restrain uterine hæmorrhage; and pressure has, I believe, been justly most valued by the accoucheur. Cold applications, in a variety of ways, have proved serviceable; but here I beg to remark, that I have known the use of the lower limbs taken away for several months by too long a continuance of cold cloths, yet not longer than the appearance of discharge seemed to warrant. The period of time during which cold cloths were applied was in one instance half an hour, in the other somewhat longer, yet I have known this period considerably exceeded, without the same bad consequences arising. Perhaps the temperature of the weather, and the peculiar constitution of the patients, might have influenced the effects; but to the impression of cold chiefly, after enduring for many hours severe labour pains, do I attribute the weakness which was experienced by these patients in their lower limbs. One was unable to stand alone for the space of three months.

The ergot of rye is a medicine of considerable utility in producing uterine contraction; but it must be given before fainting comes on, to prove serviceable. I have often administered it immediately after the birth of the child, in anticipation of flooding, where past experience has given me too much reason to expect a return: for flooding is habitual to some constitutions,—generally the weak and irritable. With scarcely an exception I have found the ergot of rye effectual in promoting uterine action. I generally administer the the tincture

* From the Madras Quarterly Medical Journal, Vol. ii.: 1840.

which has this to recommend it—it is very easily taken in any thin fluid, and is not readily injured by keeping. The emptying of the vagina and uterus of coagula is sometimes necessary, but without pressure from a bandage, which I always afterwards apply, it would not prove satisfactory. The necessity for the removal of coagula, and the redistension of the uterus, which now and then occurs, may, I think, both be prevented by the timely use of a tourniquet. The introduction of one hand within the uterus, and the application of the other without, to compress the bleeding vessels and stay hæmorrhage, has always appeared to me to be attended with much uncertainty, and some risk. Plugging the vagina is not to be depended upon, and the transfusion of human blood has not now many advocates. As immediate relief must be given, the administration of medicines, whether acids, sugar of lead, or any other drug, are of doubtful efficacy. I once saw a patient to whom a full dose of opium had been given prior to my assistance being requested by the gentleman in attendance. It was the worst case for the duration of alarming symptoms I ever witnessed, in which recovery took place. The long-continued state of exhaustion, and the frightful prostration of power, I attribute, in a very great degree, to the sedative effects of laudanum. I have often given laudanum to allay irregular and ineffectual uterine action early in labour, with beneficial results, but I have never seen it produce uterine contraction in cases of flooding. To surround the abdomen of every recently delivered woman, with a bandage capable of giving some temporary support, is in general practice, and much to be recommended, but the bandage or napkin ordinarily provided for this purpose is far from being efficient in cases of flooding. It is very easy displaced by a slight change of posture, however carefully applied, and although I have always in addition put a compress of some kind or other over the uterus, generally a pin-cushion or a small firm book enclosed in a napkin, pressure made by the hands is absolutely necessary in dangerous cases, and that to a degree painful and tiresome; before you can abate your watchful attentions and services. A bandage wide and deep, made of strong calico, with tapes attached to each side at different distances, so as to be made capable of affording support to a woman either before or after delivery, was strongly recommended about twelve or fourteen years since, by, I believe, Mr. Gaitskill, of Rotherhithe. This I have found very useful, previously placing the compress before described over the uterus. But this comes far short of the good and efficient pressure so readily to be obtained by the application of the tourniquet. This instrument is not easily displaced, as the band is passed beneath the back and over the ilia, and by turning the screw placed with the compress over the uterus, pressure is effected directly downwards upon it, and such a compression of the bleeding vessels or sinuses takes place, that uterine hæmorhage must be restrained in almost every case; indeed, as Mr. Walford said, it might be carried to such an extent as to suspend the circulation in the abdominal aorta. In thin persons, no doubt, it might effect this object; and if so, few women ought to die of flooding after delivery. I recollect, when a pupil, having been sent for the late Dr. Batty, to assist in a case of flooding, and he being unable immediately to attend, directed me to inform the gentleman in attendance that if it continued he was to place the whole weight of his body upon the patient's abdomen, by sitting astride her till he came; but the patient did well without this extraordinary help.

Mr. Walford recommended the tourniquet to be made at least double the size of the one in ordinary use, with a proportionably wide band, and this must have obvious advantages over a smaller one. The one I have hitherto used has been taken from an amputating case. I removed the pad, and by increasing the length of the fellet sufficiently to encircle the hips, with this and the compress I have obtained a power far exceeding any hitherto obtained by other means. I am disposed to think the most eligible compresses might be made of a piece of cork, about an inch in thickness, covered with soft leather, and shaped somewhat to the inferior and anterior aspect of the abdomen; this would be sufficiently firm to yield uniform pressure over the uterus without giving any pain, which I have found the corners of a book apt to do; or something softer might be applied first upon the body if thought desirable. Twice I have tried the tourniquet on the same patient in flooding labours, with satisfactory results. The first time I attended this patient, which was of her fourth child, she flooded dreadfully, and so perilous was her condition that I did not think it possible she could recover. The debility, as usual in such a case, was long continued, and so debilitating to the poor mother, that to nurse the child was out of the question. When called upon to assist at her next confinement, I was prepared with a tourniquet, and tr. secale cornut. She began to lose blood very freely after the birth of the child, the placenta being detached and lying in the vagina. I gave immediately a dose of the secale cornutum, and applied the tourniquet, shortly after which I gently removed the placenta, and all further hæmorrhage was quickly restrained by increasing the pressure; fainting did not ensue, aud I was relieved of all that bodily exertion which had been so fatiguing to me upon the former occasion.

At her subsequent labour I had to encounter the same danger of flooding, which commenced as before, and which, by the use of the tourniquet and compress *alone*, was most satisfactorily suppressed. This labour was of twelve hours' duration, and the latter pains were very severe: its progress was regular, the head of the child gradually advanced, and after its extrusion, several pains were required to bring the shoulders, body, and hips into the world. The placenta was found loose in the vagina. All this was very favourable for the non-supervention of flooding, but it did appear; and at this time I removed the placenta, which gave a momentary facility to the free discharge of blood, before I turned the screw of the tourniquet, and quickly stopped all further flow. The patient has made a much better nurse, and has been altogether stronger and better after this confinement than any former one, except her first, which occurred in the country about twelve years since. Sleep soon followed the delivery, and I left my patient with a soft compress and the tourniquet upon the body for two hours, when I returned and saw her placed comfortably in bed. She had not one bad symptom, nor yet an after pain, neither did I give one drop of laudanum.

This excellent recovery is further remarkable from the circumstance that she was seized with a very severe attack of influenza a month only before labour commenced, and when daily expecting to be confined; this seizure was attended with such an acute pain in the right side of the abdomen, upon coughing, that she was obliged herself to press a book upon the part to make it endurable. Opiates, blue pill, and Dover's powder, salines with antimony, were administered with only partial relief; and after suffering a few days, the fever being considerable, and the coughing and pain very distressing, I felt myself compelled to bleed her, which was of great service, and by continuing the antimony, in increasing doses, and the sedative at night, she was in a few days more convalescent, and gradually recruited her usual degree of strength before her accouchment. Her pregnant state, her daily expecting to be confined—indeed, she thought her labour had commenced when she sent for me, when suffering such acute pain in the side, and knowing the disposition to flood after delivery—made me most anxious to avoid taking away blood if possible. The cause of flooding in these cases is considered to be a torpor of the uterus, an exhaustion of its energy, and the consequent inefficient contraction of its fibres, and imperfect closure of the mouths of vessels where the placenta was attached; the immepiate object of the accoucheur is to put a stop to the flow of blood, and this I have succeeded in doing by the use of the tourniquet and compress; and if contraction of the uterine fibres be not at the same time induced, it must soon follow, for in proportion as the patient recovers from that state of exhaustion, be it more or less, which usually succeeds delivery, the uterus will acquire contractile power. Time and safety are gained, and relief from much bodily exertion and anxiety of mind, though not of all watchfulness. A cool room, and a quiet state of mind, if possible to be induced, will contribute to the patient's welfare, and the attendant may administer any medicine he approves, according to circumstances, or give none if he prefers.

I hope that I have not overrated the good effects of the application of the tourniquet and compress in cases of flooding after delivery, and sure I am, that if only a portion of the evils attendant upon and following these cases can be in future prevented, a great good will be obtained for many parents and infants.

I feel some desire to state, that early in my professional practice I met with rather an extraordinarry occurrence, viz., the expulsion during the last pains of labour of the whole of the uterus entire—the child, placenta, and membranes, unbroken upon the bed. At first I was a little astonished, but the momentary surprise yielded to my doing something towards saving the life of the child; I therefore immediately ruptured the membranes, discharged the liquor amnii, and separated the infant, which soon began to cry, and both it and the mother did well. I had been in the house only a very short time before I was summoned to give this assistance. The labour was quick, and took place at the full period of utero-gestation. The mother had borne one child only before, which was then twelve years old.

In the month of October last year, I had the pleasure to see the very good effects of a new midwifery instrument, (the whalebone vectis,) in the hands of Dr. Conquest, in a case of protracted labour under the care of a lamented friend. A woman, ætat. 35, strong and healthy, in labour of her first child, went on favourably for some hours; the head presented, and the os uteri became fully dilated; but notwithstanding the pains were frequent and forcible for several succeeding hours, the labour made no progress; and as various means had been tried, such as bleeding, secale cornutum, purgatives, and salines, and I think a dose of laudanum, without any increased probability of it being soon brought to a termination, it was decided in consultation that the patient should be delivered by the aid of instruments. The os uteri, I have stated, was fully dilated, the ear could be felt above the brim of the pelvis and to one side of the symphysis pubis; the fore-head was the presenting part, with the face towards the pubes; not much of the head had entered the superior aperture of the pelvis, and in the absence of pains the finger could readily be passed round it; and the vagina was so dilatable that a small hand was easily passed within it. Dr. Conquest considered the situation of the head too high up for the safe or favourable employment of the forceps, and that in all probability we should be obliged to resort to the operation of cephalatomia.

Dr. Conquest exhibited to us the whalebone vectis, which he said was the invention of a gentleman whose name I do not remember, but the maker's name is Maw, of Aldersgate Street. He strongly recommended it as a safe and useful instrument, capable of affording all the aid to be obtained by the forceps without increasing the danger. It is formed of a thin loop of whalebone affixed to a handle. This instrument was passed over the occiput with the greatest facility by Dr. Conquest, and traction was made by him and myself alternately during the presence of pains. After a few efforts the head descended a little; by persevering in the traction it was brought without the os externum, in half an hour from the time instrumental assistance commenced. Dr. Conquest had some reason to expect that, as the occiput was brought down, the face might ascend, but this did not happen: the instrument being applied to that part of the occiput adjoining the vertebræ, traction brought down the head as it presented, face and forehead first. The child was dead, as was expected, no motion having been perceptible for many hours, and the patient had been thirty in labour. The placenta was adherent to the uterus, and required manual assistance for its removal. The recovery of the patient was perfect and uninterrupted. I was much pleased to see the delivery effected so easily, and by such simple means, and I cannot do otherwise than join Dr. Conquest in recommending this very valuable instrument to those of the profession who may not have seen or heard of it.—*Ibid.*

---

*Puerperal Convulsions. Artificial Delivery of Twins.* By Edward Augustus Cory, M. D.—A German woman, named Zimmerman, of a leucophlegmatic constitution, during the process of her second parturition, was attacked with convulsions early in the morning of the 8th of June. She had been under the care of her midwife for some hours previously, and had complained during the preceding day of intense headach, for the relief of which her friends had very improperly recommended her copious potations of gin and water. On the supervention of the convulsive attacks, it was deemed expedient to procure the assistance of a medical practitioner; and Mr. Beale, surgeon, of Bedford Square East, was accordingly sent for, who immediately and very properly bled her to a considerable extent, and had also recourse to the usual secondary remedial agents. The os uteri at that time showed no signs of sufficient dilatation to permit the artificial evacuation of the uterine contents, and she almost immediately sunk into a state of complete coma. Mr. Farrer, surgeon, of the Commercial Road, had also very kindly lent his assistance. I was requested by the above gentlemen to see her about eight o'clock on the same morning. I found her in a state of complete insensibility, with no interval of consciousness. The breathing was stertorous, and the pulse so feeble as entirely to preclude any further depletion, which appeared to have been carried to its fullest extent. The os uteri was at this time dilated sufficiently to admit the careful application of the forceps, and I accomplished the delivery of the infant without difficulty. Another child was now detected in the uterus under head-presentation, which I immediately delivered by the operation of turning. The uterus showed no disposition to contract after the expulsion of the children, but by the employment of compression externally, it contracted tolerably, and one large placenta was expelled. There was no hæmorrhage: both children were still born. The woman died in about an hour after delivery.

*Sectio cadaveris*, 24 *hours after death.*—The dissection of the body was performed by Mr. Beale, assisted by Mr. Charles Bell, one of my pupils. On the removal of the dura mater, considerable vascular turgescence was observable on the surface of the left cerebral hemisphere, and some patches of imperfectly formed lymph were also evident. The tunica arachnoides presented a remarkable degree of dryness. On the superior portion of the anterior lobe, some extravasated blood was discovered, and on extending the dissection, the left ventricle was found to be completely filled with a coagulum. The effused blood, when collected, weighed altogether about three ounces. The right hemisphere partook but slightly of the increased vascularity which had been observed on the opposite side, and was comparatively healthy throughout its whole structure. Nothing further of interest was noticed in the dissection of the brain. The intestines were much distended with fœtid gas. The uterus and its appendages were in a healthy condition, and presented the appearances usually observed in a woman recently delivered.

*Remarks.*—The above is a case of the apoplectic form of puerperal convulsions, which may be considered the most fatal of all the varieties of that formidable and frightful affection. I think it may very reasonably be assumed, that if the patient, in the first instance, had been under the care of a medical practitioner, instead of a midwife, her life might have been saved—I mean, had she been copiously bled and purged on the accession of the intense headach, of which, it will be recollected, she complained during the preceding day, instead of having been plied with ardent spirits by her ignorant friends, the more formidable stage of the disease might have been effectually prevented. I do not intend to trespass on your valuable columns, by detailing the symptoms and pathology of the disease under consideration, for they can be fully comprehended by a reference to any of the standard works on obstetric medicine; I shall merely observe that some writers, among whom may be mentioned Baudelocque and others, have described several varieties of puerperal convulsion. I am, however, of opinion that the division of Dewees into hysterical, epileptic, and apoplectic, is pathologically correct, and sufficient for ordinary practical purposes. It appears that primiparous women, and those having more than one child in utero, are the most liable to puerperal convulsions, and that these attacks are more common and dangerous during parturition than at any period of utero-gestation or after delivery.—Mauriceau had 42 cases of the above disease, of which 7 occurred during pregnancy, 3 of which proved fatal; 19 during labour, of which 11 died; 16 after delivery, of which 5 died. Merriman cites 48 cases: 6 occurred after delivery; 3 during labour with twins, of which one died. The rest were attacked during labour, of which 11 were delivered by the forceps; 9 by cephalotomy, of which 2 died; 4 by version, of which 2 died; 1 died undelivered; 14 were delivered by the natural efforts, of which 5 died. Of these, 36 were primiparous. At the Maternité of Paris, under the surveillance of Madame Lachapelle, in 15,652 women delivered there, 40 were the subjects of puerperal convulsions—12 of these were delivered by the forceps; 5 by version. 23 of these cases occurred before delivery, of which 9 died. M. Pacoud, at the Maternité of Bourg, in 11,208 women, had 47 cases of this disease—18 of which occurred during pregnancy, 20 during labour, and 9 after delivery. The number of deaths is not stated in the report. M. Desjardins relates 7 cases, 5 of which happened during labour, and 2 after delivery, all of which recovered. M. Champion had 10 cases, all of which were primiparous; 7 recovered, 3 died; 5 of the children were born alive. Velpeau gives us an account of 21 cases—7 took place during pregnancy, of which 2 died; 5 during labour, of which 2 died, and 9 after delivery, of which 4 died. Collins records 19 cases, which occurred in the practice of Dr. Joseph Clark, of Dublin, of which 16 were first births. He also mentions 30 cases of his own, of which 29 were primiparous. Dr. Ramsbotham, senior, mentions 22 cases, of which 15 were first births. Of 59 cases attended by Dr. Ramsbotham, junior, 17 occurred before the commencement of labour, 28 during the process, and 14 after parturition. There were 3 cases of twins; 45 were first births; 13 of the women died. Of the children, 41 were expelled naturally by the head, 6 delivered by craniotomy, 6 by the forceps, 5 by turning, and 4 presented the breech; 23 of these only were born alive. The convulsions took place after delivery in 12 of these cases. One patient was attacked nine days after labour, another ten, and another seven.

The treatment of purperal convulsions can be comprised in a few words. Bleeding, not to ounces, but to pounds, according to the state of the patient, and delivery as soon as it can be safely accomplished. The immense quantity of blood which may be taken in this disease, with the most beneficial results, is truly astonishing. Active purgation, refrigeration of the head, counter-irritation, &c., may be regarded as useful, although of secondary importance. Opium is decidedly injurious in whatever form it may be administered, unless in the hysterical variety of the affection, where I should consider it of doubtful efficacy. Some observations appeared in a late number of the Lancet, from the pen of my able and ingenious friend, Dr. Maddock, of Judd Street, Brunswick Square, in reference to a case of puerperal convulsions, which occurred in the practice of a provincial practitioner, in which he (Dr. M.) strenuously recommended the use of opiate injections. Experience has taught me the utter uselessness, nay danger, of all the preparations of opium in puerperal convulsions; and if any practitioner should be sceptical on this point, I recommend him to peruse the interesting cases detailed by Dewees, and other eminent authorities, in relation to this subject.

The treatment of puerperal convulsions has been so graphically and impressively delineated by the late Dr. Gooch, that I cannot refrain on the present occasion from quoting his own words. "The remedies (says he) commonly recommended are antispasmodics, bleeding, and delivery. The first, general experience shows to be useless. Bleeding is then our sheet-anchor. Dr. Hamilton says, take away forty ounces at once, and if in two hours the patient is not saticfactorily better, take away forty ounces more. When I first heard Dr. Hamilton, in his lectures, deliver these instructions, I felt not a little astonished, but I can now conscientiously declare, that I have never had a patient die of puerperal convulsions, where the disease had been thus boldly treated; those who have died have been bled with a sparing hand, and to an insufficient amount. A little woman, about 18 years of age, of a spare habit, was seized with pain in her head and trembling, on which she fell down senseless. I was sent for, and soon after my arrival she became convulsed. This was the first case of the kind I had ever seen; and though the patient was not of a plethoric habit, I bled her to the amount of twenty ounces; before the bleeding was stopped, she opened her eyes and the convulsions ceased. I ordered her head to be shaved, directed cold applications to the scalp, and prescribed some brisk aperient medicine. Notwithstanding the favourable impression produced by the bleeding, which was followed by the action of the purgative, in a short time the convulsions returned; the bandage slipped off; and she lost about eight ounces of blood. The husband tied up her arm, and in great haste ran for me without his hat, and with his hands covered with blood. I went immediately, and took away about twenty ounces of blood more, and the convulsions ceased, but still the patient remained insensible. At ten o'clock at night I went to see her again, and just before my arrival she had a convulsive fit more violent than any preceding one. She had, since nine in the morning; lost forty-eight ounces of blood, and I now again bled her to the amount of thirty ounces: the convulsions ceased; in the morning she was decidedly better: in the course of the day uterine pains came on; she was delivered of a dead child, and gradually recovered. Give me the lancet, and deprive me of all other remedies, and I will do more good with it singly than with all others, deprived of this, put together."—*Ibid.*

---

*Twelve persons poisoned by the juice of Monkshood, Aconitum Napellus.* By M. Bolardini.—On the 11th of June, 1840, twelve persons suf fering from skin diseases or scurvy, with the view of gettiug rid of their complaints, swallowed each about 2 oz. 6½ drachms of the juice of monkshood, in mistake for that of scurvy-grass. An old man, about sixty years of age, was the first to suffer; his respiration became impeded, and he was seized with vomiting. A physician who was sent for mistook his complaint, and he died in a few hours.] Two women, each about fifty-five years of age, and the subjects of scurvy, became uneasy soon after swallowing the poisonous dose; convulsions, with prostration of strength, and paralytic loss of power of limbs, soon made their appearance, and they both died about two hours after partaking of the poison.

The other nine individuals were all violently affected, and would have fallen victims to the poison if relief had not been speedily administered.

The symptoms experienced were the following: extreme prostration of the strength of the body as well as of the powers of the mind; the countenance was very pale, and its expression much altered: the pupils of the eye were greatly dilated; the eyes had lost their lustre, and were surrounded by a bluish circle; vertigo, and headach, especially severe towards the occiput, were complained of; the abdomen was much swollen and painful; they all vomited greenish matters, and a few suffered from diarrhœa, the matters passed being of a similar colour. Feelings of oppression and great anxiety were experienced by all, together with a general sensation of cold, which increased rapidly with the poisonous symptoms; the nails were of a livid colour; cramps were complained of in the legs; and the pulse was small, feeble, and scarcely perceptible.

Emetics were first administered to excite vomiting; after which tonics and stimulants were freely given, as tincture of canella, anisette, rum, wine, &c., carried the length of producing intoxication; whilst, at the same time, the limbs were freely stimulated by frictions with spirituous lotions. Under this treatment the nine cases recovered.

The following morbid appearances presented in the three fatal cases. No change was observed to have occurred in the surface of the bodies. The vessels of the *pia mater* and arachnoid membranes were much injected with blood, and a large quantity of serous fluid was found under the arachnoid membrane, and at the base of the brain. There was, however, no effusion of blood in the ventricles of the brain. The lungs were gorged with blood; the heart was flabby, and contained black blood, and the large vessels were distended with the same. The liver and spleen were healthy. The stomach was distended with gaseous matter, and contained a quantity of viscid ash-gray coloured liquid. Irregular injected patches were observed scattered in detached points over the lining membrane of the stomach, especially towards its larger curvature. The duodenum and small intestines presented here and there red patches, and contained matters of the same colour and quality as those observed in the stomach.—*Ed. Med. and Surg. Journal, from Memoriale della Medicina Contemporanea.* September, 1840.

---

*On the Hydrated Peroxide of Iron as an antidote for Arsenic.* By M. Orfila.—In a recent case of poisoning by arsenic in Paris, the physician administered colcothar, or the anhydrous oxide of iron, instead of the hydrated peroxide: no relief whatever was experienced, and the unfortunate patient died. M. Orfila, with the view of determining the merits of these two preparations, made a number of experiments, from which it results that the colcothar, or anhydrous oxide of iron, possesses no antidotal power whatever, as it requires twelve ounces and a half of it to neutralize a single grain of arsenious acid.

It appears, however, to be proved that the hydrated peroxide of iron does to a certain extent act as an antidote to arsenious acid; and many experimentalists have shown that, if a sufficient quantity of this substance be given, the poisonous symptoms caused by arsenic are removed. It has been doubted how much of this effect was owing to the peroxide possessing the properties of a chemical antidote, or whether it merely acted by its mechanical properties of enveloping the arsenic, and preventing its acting on the stomach. That it possesses the properties of a chemical antidote, the researches of Guibourt, Deville, Nonat, Sandras, and of Dr. A. D. Maclagan, abundantly prove; at least they prove that arsenic is removed from its solution in water by the hydrated peroxide of iron forming a chemical compound, apparently insoluble in water. The quantity of the peroxide required to produce this effect varied greatly in the experiments of these gentlemen; for whilst Dr. Maclagan found that twelve parts sufficed to neutralize one of arsenic, Guibourt found that about sixty were needed.

M. Orfila, admitting the correctness of the statement as to the hydrated peroxide of iron entering into chemical combinations, and forming with arsenic a compound insoluble in water, performed a number of experiments, for the purpose of ascertaining whether this apparently insoluble compound was not liable to be acted upon by the juices of the stomach, and still produce poisonous symptoms, as MM. Nonat, Deville and Sandras had previously announced that the compound was still poisonous. M. Orfila, therefore, administered to several strong dogs, seventeen grains of arsenic, with eight drachms of the dried hydrated peroxide of iron. This was allowed to form a chemical combination previous to its administration, so that not a particle of free arsenic could be discovered in it, and it admitted of being boiled in water without imparting to that fluid the slightest trace of the poison. In all the dogs, copious alvine evacuations were induced, together with the usual symptoms of poisoning by arsenic. They all died in from 28 to 40 hours, provided vomiting was prevented; and on examining their bodies, arsenic was detected in the urine and liver. The alimentary canal, however, presented scarcely any marks of inflammation.

From these experiments, M. Orfila concluded that the dry hydrated peroxide of iron absorbs and neutralizes a considerable quantity of arsenious acid, forming with it a sub-arsenite of iron, still poisonous, but less so than arsenious acid itself; that the poisonous effects of this compound are apparently due to its being decomposed by the acid contents of the stomach, which set free the arsenious acid, and allow of its being absorbed; that it is, therefore, a wise measure, in cases of poisoning by arsenic, to administer the dry hydrated peroxide of iron, after having mixed it with water, and then to excite vomiting, which both expels the arsenic itself from the stomach, and the sub-arsenite of iron formed there; that, provided a sufficiently large dose of hydrated peroxide of iron has been administered, no fears need be entertained of the decomposition of the sub-arsenite of iron in the stomach, even though vomiting should not have been excited, as, in proportion as the acids of the stomach decompose one portion of the compound, the arsenious acid set free is immediately neutralized, and enters into combination with another portion of the ferruginous oxide. That, in every case, therefore, the dose of the peroxide ought to be very large.

As all the peroxide of iron contains in natural combination a certain proportion of arsenic, it might easily happen that in a case of suspected poisoning by arsenic, and where the peroxide had been administered as an antidote, this accidental occurrence might complicate the inquiry. M. Orfila, with his usual ability, has, however, furnished us with the means of ascertaining on chemical analysis, what portion of the arsenic found has been derived from the poison in the stomach, and what portion from that naturally contained in the peroxide. The peroxide rejected by vomiting, or found in the stomach, is boiled in distilled water for twenty-five or thirty minutes, and if any free arsenious acid exists, it will be dissolved, and found in the aqueous fluid. If none, however, be detected, three or four drachms of the washed peroxide, with as much pure potash, must be agitated in a vial with alcohol, in the cold, for some time. This alkali, which does not act upon a single atom of the arsenic naturally contained in the iron, combines, nevertheless, with the arsenious acid of the compound which has been found in the stomach, provided it exists in any appreciable quantity. The usual tests applied to the alkaline solution will easily show whether or not any arsenic has been present. If the peroxide, however, be boiled in the alcoholic solution of potass, the whole of the arsenic which is contained in the peroxide is dissolved, both that which exists naturally in it, and that which may have resulted from the formation of a sub-arsenite in the stomach. —*Ib., from Bul. de l'Acad. Roy. de Med.*, 16th March, 1841.

---

*Symptoms of Poisoning produced by the endermic application of the Acetate of Morphia.* By M. Dupont.—Madame C., 25 years of age, and with disease of the heart, on account of acute pain at the margin of one of the lower right ribs, had a small blister applied, and the blistered surface afterwards powdered with about four-tenths of a grain of the acetate of morphia. In about twenty minutes after the application of the morphia, she complained of indistinctness of vision, which was soon followed by vomiting and delirium. She attributed those symptoms to the plaster which was spread over the morphia, and called for its instant removal, which was done. She was seen by her medical attendant a few minutes afterwards. She was vomiting every now and then whitish matters; her pulse was extremely weak, her face pale, the eyes sunk, the pupils dilated; the superior and inferior extremities in a complete state of relaxation, and she had slight delirium. Sinapisms were applied, and drinks containing vinegar and ether were administered. The vomiting, however, still continued; decoction of nut-galls was therefore given every quarter of an hour, and cold gum water, but without affording any relief. Four hours elapsed, and the pulse became so feeble as scarcely to be felt; the face became covered with a slight sweat; the voice was almost lost, and the respiration stertorous and interrupted. Infusion of coffee, alternated with eau sucrée, was given, and continued for another hour without affording relief. The anti-emetic potion of Riviere was then twice given, which had the effect of stopping the vomiting. Slight incoherence continued during all the succeeding day, but by the day after no traces remained of the poison.—*Ib., from Jour. de la Soc. de Bourdeaux*, January, 1841.

---

*Case of Secretion of Air from the Human Skin.* By Sir Francis Smith.—An hypochondriac gentleman, 35 years of age, informed Sir F. Smith that he was liable to immense disengagements of gas from the stomach; that he also occasionally discharged air from the urinary bladder, and had observed an escape of air from the surface of his body when under water in the bath. Little attention was paid to the last of these statements till the 15th of May, 1840, when Sir F. Smith was hastily summoned to the bath to see the phenomena of the disengagement of air from the skin of his patient. He was found in a bath at 79°, and his chest, abdomen, shoulders, and hands were literally covered with small air bubbles. When he removed his hands from the bath, the bubbles disappeared, but when he replaced them below the water, the air bubbles were observed to make their re-appearance, at first very minute, but gradually increased in size till the palms of his hands became again coated with them. He frequently wiped away the bubbles from his hands and chest, but in every case they were soon replaced by others. The bubbles of air ran together, when pushed with the finger, like globules of mercury, without quitting the skin, or becoming loose in the water. This circumstance was observed for twenty minutes by Sir F. Smith, and towards the end of that time the margins of the upper end of the bath opposite where the shoulders had been, were coated all around for a depth of about two inches, with minute bubbles of air.—*Dublin Journ. of Med. Sci.*

---

*Extraction of a foreign body from the Uterus.* By M. Bocande.—A woman, of about 30 years of age, applied to the Hospital of St. Louis on account of an uterine affection. She related that she had become pregnant when 28 years of age, but had miscarried at the fifth month, and remained for upwards of three months afterwards in bed in consequence of inflammation of the uterus. She had sought relief at another hospital, but after a resideuce there of several months, was dismissed without being cured. She had a pale sickly unhealthy look; complained of indigestion, and of dull continued pain in the lumbar and hypogastric regions, had an accession of hectic fever every evening. An irregular hard tumour filled the pelvis, and projected into the right iliac fossa. On examination with the aid of the speculum, the uterine orifice was seen half open, but the lips natural. Within the uterus was seen a white body, which, when touched with a stilet, gave a ligneous resistance, but permitted the instrument to be passed over it before and behind, but appeared at its lateral extremities to be fixed in the coats of the uterus. M. Maisonneuve attempted to cut this body through with a pair of long scissors, that it might be more easily extracted, but could not effect it. He then seized it with a pair of polypus forceps, and by firm but gentle traction, disengaged first one extremity, and then the other. It was a peg of wood pointed at one extremity, and as it were, twisted at the other.

M. Maisonneuve supposed that this piece of wood had been used for the purpose of producing abortion, but had become engaged in the parietes of the uterus after having effected its purpose; and subsequent inquiries confirmed this supposition.

The woman recovered, but the tumour still remained in the pelvis, probably the consequence of adhesions between the uterus, bladder, rectum, and some folds of intestine.

*Gaz. Med.*

